# Amino acid transporter CsBAT links GABA accumulation to flavonoid metabolism in *Camellia sinensis*

**DOI:** 10.1093/hr/uhaf261

**Published:** 2025-10-01

**Authors:** Lin Feng, Panpan Liu, Yuanyuan He, Shengpeng Wang, Rui Luo, Anhui Gui, Jinjin Xue, Shiwei Gao, Pengcheng Zheng

**Affiliations:** Key Laboratory of Tea Resources Comprehensive Utilization, Ministry of Agriculture and Rural Affairs, Hubei Qingzhuan Tea Engineering Research Centre, Wuhan, Hubei 430064, China; Fruit and Tea Research Institute, Hubei Academy of Agricultural Sciences, Wuhan, Hubei 430064, China; Key Laboratory of Tea Resources Comprehensive Utilization, Ministry of Agriculture and Rural Affairs, Hubei Qingzhuan Tea Engineering Research Centre, Wuhan, Hubei 430064, China; Fruit and Tea Research Institute, Hubei Academy of Agricultural Sciences, Wuhan, Hubei 430064, China; Fruit and Tea Research Institute, Hubei Academy of Agricultural Sciences, Wuhan, Hubei 430064, China; College of Horticulture and Forestry Sciences, Huazhong Agricultural University, Wuhan, Hubei 430070, China; Key Laboratory of Tea Resources Comprehensive Utilization, Ministry of Agriculture and Rural Affairs, Hubei Qingzhuan Tea Engineering Research Centre, Wuhan, Hubei 430064, China; Fruit and Tea Research Institute, Hubei Academy of Agricultural Sciences, Wuhan, Hubei 430064, China; Key Laboratory of Tea Resources Comprehensive Utilization, Ministry of Agriculture and Rural Affairs, Hubei Qingzhuan Tea Engineering Research Centre, Wuhan, Hubei 430064, China; Fruit and Tea Research Institute, Hubei Academy of Agricultural Sciences, Wuhan, Hubei 430064, China; Key Laboratory of Tea Resources Comprehensive Utilization, Ministry of Agriculture and Rural Affairs, Hubei Qingzhuan Tea Engineering Research Centre, Wuhan, Hubei 430064, China; Fruit and Tea Research Institute, Hubei Academy of Agricultural Sciences, Wuhan, Hubei 430064, China; Key Laboratory of Tea Resources Comprehensive Utilization, Ministry of Agriculture and Rural Affairs, Hubei Qingzhuan Tea Engineering Research Centre, Wuhan, Hubei 430064, China; Fruit and Tea Research Institute, Hubei Academy of Agricultural Sciences, Wuhan, Hubei 430064, China; Key Laboratory of Tea Resources Comprehensive Utilization, Ministry of Agriculture and Rural Affairs, Hubei Qingzhuan Tea Engineering Research Centre, Wuhan, Hubei 430064, China; Fruit and Tea Research Institute, Hubei Academy of Agricultural Sciences, Wuhan, Hubei 430064, China; Key Laboratory of Tea Resources Comprehensive Utilization, Ministry of Agriculture and Rural Affairs, Hubei Qingzhuan Tea Engineering Research Centre, Wuhan, Hubei 430064, China; Fruit and Tea Research Institute, Hubei Academy of Agricultural Sciences, Wuhan, Hubei 430064, China

## Abstract

γ-Aminobutyric acid (GABA), a four-carbon non-protein amino acid functions as a key signaling molecule in plants. As a signature bioactive compound in tea, GABA plays a crucial role in determining both flavor profile and health-promoting properties. Despite its importance, the molecular regulation of GABA accumulation in tea plants—especially its metabolic crosstalk with key quality determinants like flavonoids—remains elusive. While amino acid transporters are known to mediate source–sink allocation in plants, the functional characterization of GABA transporters in *Camellia sinensis* has been lacking. In this study, we identified and functionally characterized the bidirectional amino acid transporter CsBAT in tea plants. Through a comprehensive multiplatform validation system encompassing yeast heterologous expression, *Arabidopsis* genetic transformation, and tea transgenic system, we revealed that *CsBAT* shows vascular-specific expression and facilitates directional amino acid transport from source (mature leaves) to sink (young shoots), thereby significantly boosting GABA accumulation in buds and young leaves. Importantly, we discovered that CsBAT functionally interacts with key flavonoid biosynthetic enzymes (LAR, 4CL, C4H) within secondary metabolic networks. Our findings provide the first mechanistic link between *CsBAT*-mediated amino acid transport and tea quality formation, establishing both theoretical frameworks and practical tools for molecular breeding of premium tea cultivars.

## Introduction

Tea plant (*Camellia sinensis*. L), an important leaf-harvested crop, accumulates various amino acids in its buds and young leaves that significantly influence tea flavor and health benefits [1, 2]. Theanine, glutamine, and γ-aminobutyric acid (GABA) serve as crucial nitrogen storage forms, playing pivotal roles in nitrogen recycling while bridging primary metabolism with secondary metabolic pathways like flavonoid biosynthesis [3, 4]. GABA, a nonprotein amino acid synthesized in the cytosol via glutamate decarboxylase (GAD)-mediated decarboxylation of glutamate, is subsequently transported into mitochondria where GABA transaminase (GABA-T) converts it into succinic semialdehyde for TCA cycle entry [5]. As a characteristic secondary metabolite, GABA contributes significantly to tea’s unique sensory properties and health-promoting effects [6, 7]. Tea containing ≥1.5 mg/g GABA (designated Gabaron tea) exhibits notable antihypertensive, antidepressant, and neuroprotective properties [8–10]. Current GABA enhancement methods (e.g. anaerobic treatment) often compromise tea quality by inducing off-flavors and disrupting flavonoid homeostasis, highlighting the need for genetic approaches to modulate GABA accumulation [10–13].

GABA distribution in plants is strictly regulated by amino acid transporters [14–16]. Long-distance amino acid transport via vascular systems involves complex transmembrane processes mediated by two major transporter families [17–19] s: amino acid transporters (ATF) and amino acid/polyamine/organocation (APC) transporters [20, 21]. While microbial GABA transport is primarily mediated by APC transporters [22, 23], *Arabidopsis* high-affinity transporters *AtGAT1* (plasma membrane) and *AtGABP* (mitochondrial) have been characterized [5, 24]. Notably, *AtGABP* shows bidirectional transport activity and regulates carbon–nitrogen balance, but GABA transporters in tea plants remain understudied [18, 24].

Our preliminary studies identified *CsBAT*, a tea transporter homologous to microbial GABA transporters, demonstrating GABA transport activity in yeast with expression positively correlated with leaf maturation (*P* < 0.01). We hypothesize that *CsBAT* mediates GABA translocation from mature leaves (source) to young shoots (sink), dynamically regulating GABA accumulation. Given flavonoids’ importance as key quality determinants and emerging evidence of GABA’s role in modulating flavonoid metabolism via carbon skeleton provision, calcium signaling, and PAL regulation, we propose that *CsBAT* mediates GABA–flavonoid crosstalk to influence tea quality. Using an integrated approach combining yeast complementation, tea transgenic system, and metabolic profiling, this study will elucidate *CsBAT*’s mechanistic role in linking GABA transport with flavonoid biosynthesis, providing novel molecular targets for tea quality improvement.

## Results

### CsBAT is a vascular-localized transporter

**Figure 1:**
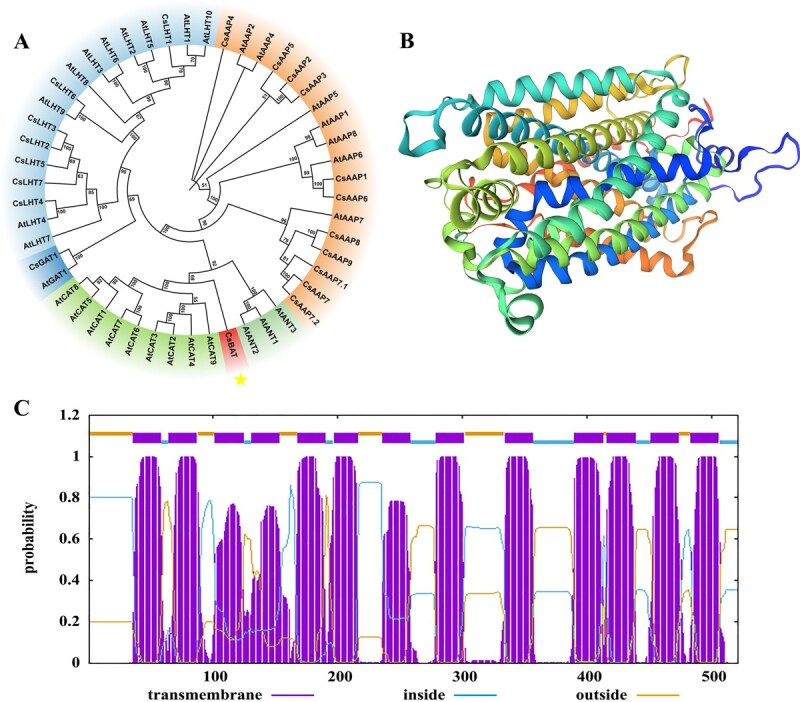
Phylogenetic tree, structure analysis and transmembrane domain analysis of CsBAT. (A) Phylogenetic tree analysis of CsBAT. (B) The 3D structure modeling of the CsBAT protein. The structure was producted via the Phyre2 website (http://www.sbg.bio.ic.ac.uk/phyre2/html/page.cgi?id=index). (C) Prediction of the transmembrane domains of CsBAT using the TMHMM-2.0 website (https://services.healthtech.dtu.dk/services/TMHMM-2.0/).

We cloned the CsBAT gene (GenBank accession: KY709678.1) from tea plant (*C. sinensis*) leaves, which encodes a 533-amino-acid protein with 13 transmembrane domains (TMDs) (Fig. 1). Domain analysis revealed that CsBAT contains the 2A0303 domain (PFAM cl45918), a conserved motif associated with transmembrane transport of ammonium, amino acids, peptides, and amines (Fig. 1C). Phylogenetic analysis demonstrated that CsBAT belongs to the cationic amino acid transporter (CAT) family, showing the closest evolutionary relationship with plant CAT homologs (Fig. 1A). The Phyre2-predicted 3D structural model (99% confidence) further supported its transmembrane transport function, exhibiting a typical ‘LeuT-like’ fold characteristic of APC superfamily transporters (Fig. 1B).

**Figure 2:**
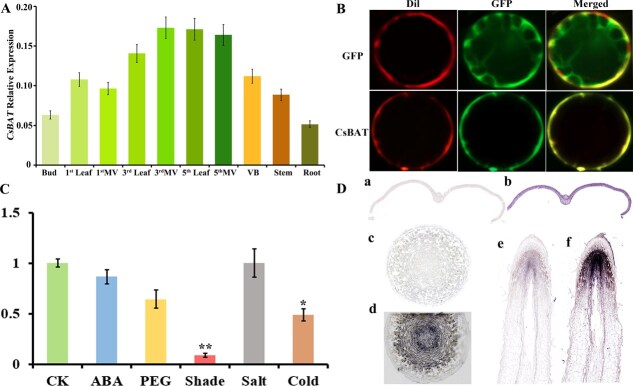
Expression patterns and stress responses of *CsBAT* in tea plants. (A) Tissue-specific expression of *CsBAT* across different organs, including buds (Bud), first leaves (1st Leaf) and their main veins (1st MV), third leaves (3rd Leaf) and their main veins (3rd MV), fifth leaves (5th Leaf) and their main veins (5th MV), stem vascular bundles (VB), stems (Stem), and roots (Root). (B) Subcellular localization of CsBAT-GFP fusion protein in *Arabidopsis* epidermal cells, showing membrane staining (DiI, left), GFP signal (middle), and merged channels (right). (C) *CsBAT* expression changes under abiotic stresses (ABA, drought, low light, salt, and cold). (D) *In situ* hybridization of CsBAT transcripts in buds (a, sense control; b, antisense signal), stems (c, sense control; d, antisense signal), and roots (e, sense control; f, antisense signal) using DIG-labeled probes. Data were analyzed using one-way ANOVA, ^*^P < 0.05, ^**^P < 0.01

To elucidate the biological function of *CsBAT*, we analyzed its spatial expression patterns in tea plants. *CsBAT* exhibited constitutive expression across all tissues, with notably high transcript levels (Fig. 2A). Notably, *CsBAT* was predominantly localized in the vascular tissues of leaves, where its expression increased with leaf maturity—significantly higher in fifth-leaf veins (5th Leaf) and third-leaf veins (3rd Leaf) compared to young shoots (Fig. 2B). Upregulation under nitrogen deficiency implies roles in nutrient remobilization and downregulation under low light, drought, and cold stress indicates the function of CsBAT in adaptive responses to maintain growth homeostasis (Fig. 2C). *In situ* hybridization further revealed ubiquitous expression of *CsBAT* in leaf tissues, with strong signals detected in stem vascular bundles and pith, root meristems and conducting tissues (Fig. 2D). Vascular-enriched expression suggests *CsBAT* regulates source-to-sink allocation of amino acids (e.g. GABA, theanine) from mature leaves to young shoots, contributing to flavor compound accumulation.

### CsBAT exhibits distinct transport affinities for GABA and other amino acids

**Figure 3:**
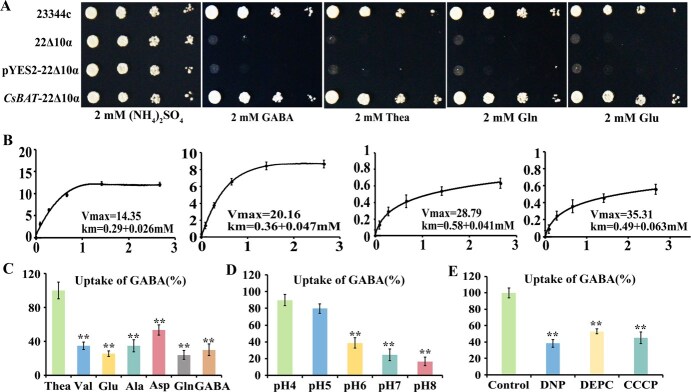
Functional characterization and kinetic analysis of *CsBAT* in the 22Δ10α yeast strain. (A) Expression of *CsBAT* rescued the 22∆10α phenotype on medium with 2 mM γ-Aminobutyric acid，2 mM glutamine, 2 mM glutamic acid, and theanine as the sole nitrogen source, with the (NH_4_)_2_SO_4_ treatment as a control of nitrogen source. The 23344c WT and 22Δ10α mutant served as positive and negative controls, respectively, pYES2 was the empty vector. (B) Absorption kinetics curve of CsBAT for Aminobutyric acid, theanine, glutamic acid and glutamine. (C–E) Effect of Hydrogen Ion Inhibitor, different pH, other amino acids on the Absorption of γ-Aminobutyric acid by the CsBAT. Data represent means ± SD (*n* = 3). Data were analyzed using one-way ANOVA, ^*^P < 0.05, ^**^P < 0.01.

To characterize the transport properties of CsBAT in tea plants, we employed a yeast complementation system using various amino acids as the sole nitrogen source. Growth assays demonstrated that only WT and CsBAT-expressing yeast could proliferate in media containing GABA, theanine, glutamate, or glutamine as the exclusive nitrogen source, while empty vector (pYES2)-transformed and mutant 22Δ10α yeast strains showed impaired growth (Fig. 3A). These results confirm CsBAT’s ability to transport these amino acids.

Kinetic analysis revealed that CsBAT displayed the highest affinity for GABA (Km = 0.29 ± 0.02 mM), followed by theanine (0.36 ± 0.03 mM), glutamine (0.40 ± 0.04 mM), and glutamate (0.58 ± 0.05 mM) (Fig. 3B). Competitive transport assays showed that the presence of xylem-mobile amino acids (glutamate, glutamine, aspartate, alanine, or valine) at 10-fold the concentration of GABA reduced GABA transport efficiency by 46.38%–75.98%, with glutamine exhibiting the strongest inhibitory effect (Fig. 3C). This suggests overlapping binding sites for these substrates on CsBAT.

The transporter activity was markedly pH-dependent, with optimal GABA uptake observed under acidic conditions (pH 4.0–5.5; inhibition rate 10.34%–20.30%). In contrast, transport efficiency decreased dramatically under neutral/alkaline conditions (pH 7.0–8.5; inhibition rate 61.39%–83.38%) (Fig. 3D). Furthermore, treatment with proton gradient disruptors (DNP, CCCP, DEPC) reduced GABA transport by 46.98%–61.64%, confirming that CsBAT operates via an H^+^-electrochemical gradient-dependent symport mechanism (Fig. 3E).

### CsBAT modulates carbon partitioning in *Arabidopsis* by affecting photosynthetic parameters and root–shoot growth balance

**Figure 4:**
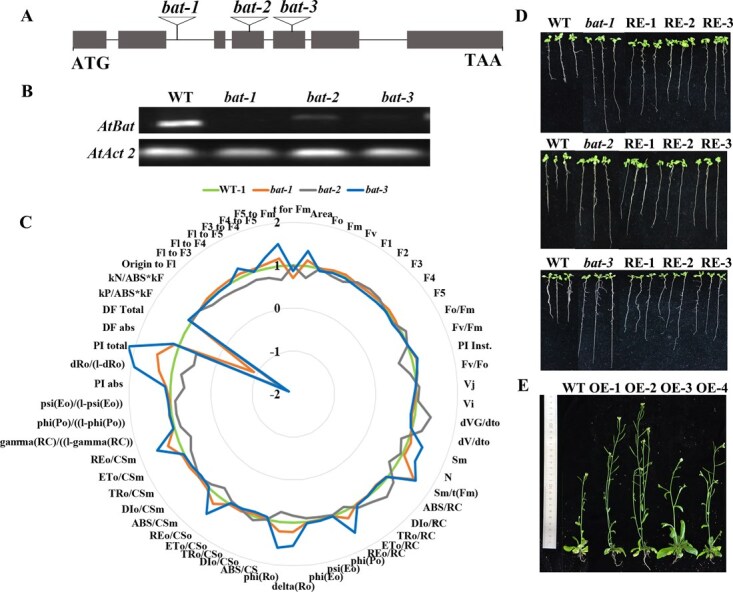
Molecular characterization and phenotypic analysis of *Arabidopsis bat* mutants and CsBAT-overexpressing lines. (A) Schematic diagram of T-DNA insertion sites in the *AtBAT* (At2g01170) gene. Exons are represented by gray boxes, and black triangles indicate the insertion positions of three SALK T-DNA mutants (*bat-1*: SALK_048889c; *bat-2*: SALK_048892c; *bat-3*: SALK_107641c). (B) Expression analysis of *AtBAT* in bat mutants. Transcript levels were determined by qRT-PCR, with values normalized to the WT (Col-0). (C) Radar chart comparing chlorophyll fluorescence parameters between WT (Col-0) and bat mutants. (D) Root growth phenotypes of different *Arabidopsis* lines grown on one-half MS medium for 10 days. (E) Growth phenotypes of 40-day-old *CsBAT*-overexpressing lines compared to WT controls.

To investigate CsBAT function, we generated complementation and overexpression lines by introducing the CsBAT CDS into expression vector pK7FWG2 via Gateway technology, followed by *Agrobacterium*-mediated floral dip transformation of *Arabidopsis bat* mutants (*bat-1*/SALK_048889c, *bat-2*/SALK_ 048892c, *bat-3*/SALK_107641c), and WT *Columbia-0* plants (Fig. 4A). Resistant transformants with varying expression levels were selected and validated by qPCR (Fig. 4B).

Chlorophyll fluorescence analysis revealed distinct differences in photosynthetic systems between WT, mutant, and complementation lines. OJIP curve analysis showed that complementation lines exhibited intermediate phenotypes between mutants and WT (Fig. S3), suggesting partial functional restoration. Radar chart analysis demonstrated that *Arabidopsis bat* mutants displayed a unique reorganization of photosynthetic parameters compared to WT: increased photosystem performance (PI total/PI Inst), PSII reaction center activity (Fv/Fo, φ(Po)), electron transport efficiency per reaction center (REo/CSm), and system stability parameters (Sm/N), but decreased overall electron transport capacity (DF Total/dVG/dto) (Fig. 4C). These results indicate that CsBAT deficiency triggers a ‘streamlined optimization’ of the photosynthetic system, characterized by reduced total reaction centers but enhanced activity and efficiency of remaining centers.

Phenotypic analysis showed that mutants exhibited significantly longer roots than WT on one-half MS medium, while complementation lines displayed intermediate root lengths (Fig. 4D). *CsBAT* overexpression lines showed increased plant height (Fig. 4E) and superior shoot growth compared to WT controls. Biomass measurements confirmed significantly higher shoot dry weight in overexpression lines **(**Fig. S4). Collectively, these findings suggest that CsBAT regulates source–sink nutrient allocation balance by promoting shoot biomass accumulation while suppressing root elongation.

### CsBAT modulates the balance of flavonoid and amino acid metabolism

**Figure 5:**
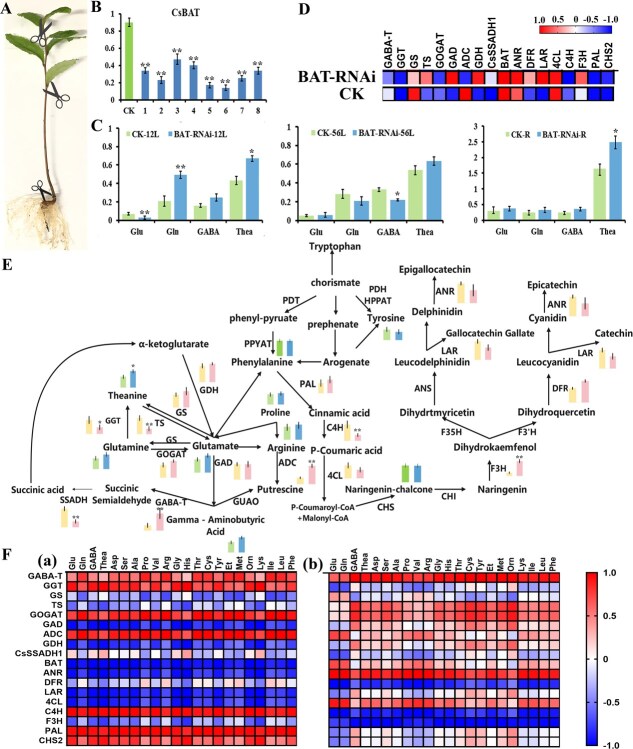
Integrated analysis of metabolic regulation by *CsBAT-RNAi* in tea plants. (A) Phenotype of tea plants with transgenic hairy roots after infection. (B) Relative expression levels of *CsBAT* in transgenic hairy roots. Plants transformed with an empty vector were used as controls. Data were analyzed using one-way ANOVA, ^*^P < 0.05, ^**^P < 0.01. (C) Comparison of amino acid content between RNAi lines and controls in different tissues (young leaves, mature leaves, and roots). (D) Correlation network analysis between *CsBAT* and genes involved in amino acid and flavonoid metabolism. Red and blue boxes represent positive (*r* > 0) and negative (*r* < 0) Pearson correlation coefficients, respectively. Boxes in contrasting colors represent positive (r > 0) and negative (r < 0) Pearson correlation coefficients, respectively. The color scale indicates the range of correlation coefficients from −1.0 (blue) to +1.0 (red). (E) Expression profiles of key metabolic pathway genes and abundance of major amino acids in RNAi lines and controls. Metabolite levels are distinguished by color coding for control and RNAi lines; gene expression levels are represented by distinct color coding for control and RNAi lines. Metabolite levels are color-coded with green for the control line and blue for the RNAi line; gene expression levels are represented in pink for the control line and yellow for the RNAi line. Data were analyzed using one-way ANOVA (^*^P < 0.05, ^**^P < 0.01). (F) Correlation analysis between metabolic gene expression and amino acid content in CsBAT-RNAi-positive hairy roots (a) and control seedlings (b). Red and blue boxes Boxes in contrasting colors indicate positive and negative correlations, respectively. All data represent mean ± SD of three biological replicates

We constructed the *CsBAT*-RNAi plant expression vector and infected tea plant hairy roots, obtaining eight positive hairy root lines with BAT relative expression levels ranging from 0.17 to 0.47 (Fig. 5A and B). In the roots, amino acids such as GABA, Glu, Gln, and Thea showed an accumulation trend in different positive hairy roots, with total free amino acid content increasing by 20%–76% (Fig. 5C). Additionally, we observed a 30%–63% increase in total free amino acids in young shoots (first and second leaves), whereas mature leaves (fifth and sixth leaves) exhibited an 8.3%–15% reduction (Fig. 5C). Combined with the high expression of *CsBAT* in the veins of the fifth and sixth leaves and its *in situ* hybridization results, we speculate that *CsBAT* primarily unloads amino acids into the phloem in roots for transport to aerial tissues to support growth. In contrast, in aboveground tissues, *CsBAT* may function in unloading and transporting amino acids from mature leaves to young shoots.

In *CsBAT*-RNAi-positive hairy roots, the expression level of *CsBAT* exhibited showed a strong positive correlation with that of *CsLAR* (*r* = 0.973), *CsGAD* (*r* = 0.961), *CsANR* (*r* = 0.949), *Cs4CL* (*r* = 0.939), and *CsGDH* (*r* = 0.890), but a strong negative correlation with *CsC4H* (*r* = −0.999), *CsPAL* (*r* = −0.960), and *CsGGT* (*r* = −0.913) (Fig. 5D). In control seedlings, the expression of *CsBAT* was positively correlated with *CsADC* (*r* = 0.999), *4CL* (*r* = 0.937), and *CsGS* (*r* = 0.926), but negatively correlated with *CsGGT* (*r* = −1), *CsC4H* (*r* = −0.999), *CsCHS* (*r* = −0.969), *CsPAL* (*r* = −0.969), *CsLAR* (*r* = −0.967), *CsGDH* (*r* = −0.895), and *CsDFR* (*r* = −0.739). Notably, the expression of *Cs4CL* consistently positively correlated with that of *CsBAT* in both groups, whereas *CsC4H* and *CsGDH* showed antagonistic (negative) expression. Interestingly, the correlation between CsLAR and CsBAT was positive in RNAi-positive roots but negative in controls (Fig. 5D).

Subsequently, we examined the expression of key genes in the GABA metabolism, theanine metabolism, and flavonoid biosynthesis pathways (Fig. 5E). In *CsBAT*-RNAi-positive hairy roots, *CsBAT* expression levels showed a significant negative correlation with the accumulation of all free amino acids, including GABA (*r* = −0.999), Glu (*r* = −0.998), Thea (*r* = −0.980), and Gln (*r* = −0.917) (Fig. 5Fa). In contrast, in control tea seedlings, *CsBAT* expression was positively correlated with amino acid accumulation. Notably, the contents of GABA, Thea, Gln, and Glu were strongly positively correlated not only with key enzymes in amino acid metabolism (*CsGABA-T*, *CsGGT*, *CsGOGAT*, and *CsADC*) but also with flavonoid pathway genes (*CsC4H*, *CsPAL*, and *CsCHS*) (Fig. 5Fb). In both *CsBAT*-RNAi and control tea seedlings, *CsGABA-T*, *CsGOGAT*, *CsADC*, *CsSSADH*, *CsF3H*, *CsPAL*, and *CsCHS* exhibited consistent correlations with amino acid levels. However, in *CsBAT*-RNAi seedlings, *CsTS*, *CsGAD*, *CsGDH*, *CsANR*, and *Cs4CL* expression was negatively correlated with free amino acid content, whereas *CsGGT* and *CsC4H* showed positive correlations. Intriguingly, in control seedlings, *CsTS*, *CsGAD*, *CsGDH*, *CsANR*, and *Cs4CL* expression was positively correlated with free amino acids, while *CsGGT* and *CsC4H* were negatively correlated (Fig. 5F). This completely opposite correlation pattern suggests that *CsBAT* knockdown alters or even reverses the expression of key genes and enzymes in amino acid and flavonoid metabolic pathways. Based on these findings, we propose that flavonoid pathway genes (CsLAR, Cs4CL, and CsC4H) may mediate BAT’s role in the transport, distribution, and metabolism of GABA in tea plants.

## Discussion

**Figure 6:**
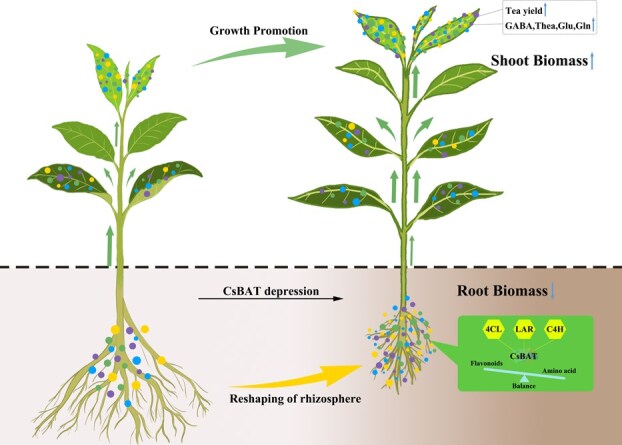
Schematic model of coordinated regulation between CsBAT and flavonoid pathway genes in modulating tea quality and yield

GABA is a key determinant of tea quality, contributing not only to the umami and sweetness of tea infusion while reducing bitterness but also regulating carbon–nitrogen metabolism to promote the synthesis and transport of secondary metabolites [1, 3, 5, 6]. Additionally, it enhances the ability of young shoots to withstand stresses such as low temperature and drought [5, 23]. This study reveals that the tea bidirectional amino acid transporter *CsBAT* exhibits high-affinity transport activity for GABA, theanine, glutamate, and other amino acids. For the first time, we demonstrate that *CsBAT* serves as a critical hub linking primary metabolism (GABA/theanine synthesis) and secondary metabolism (flavonoid pathway) by dynamically regulating the ‘source–sink’ translocation network of amino acids (Fig. 6). The comparable phenylalanine levels in the roots of RNAi and control strains suggest that the disruption of flavonoid metabolism occurs downstream of this precursor (Fig. 5C).

### CsBAT-mediated amino acid allocation favors young shoots

*CsBAT* is specifically highly expressed in the vascular tissues of tea plants, particularly in ‘one bud and three leaves’ and ‘one bud and five leaves’, suggesting its preferential role in nutrient allocation to young tissues. Using hairy root systems and RNAi, we found that *CsBAT* suppression reduced amino acid content in mature leaves (5th–6th leaves) by 8%–15%, while significantly increasing amino acid levels in young shoots (30%–63%). This confirms that *CsBAT* mediates directional amino acid transport from mature to younger leaves, facilitating GABA enrichment in metabolically active tissues—consistent with tea plants’ nutrient prioritization strategy. Furthermore, *CsBAT*-overexpressing *Arabidopsis* plants exhibited increased shoot biomass (with inhibited root growth) and enhanced photosynthetic efficiency, indicating that *CsBAT* balances source–sink relationships and aboveground–belowground nutrient competition. This ‘shoot-first’ strategy may help tea plants rapidly capture light resources under limited nutrient conditions, aligning with our observations of altered photosynthetic parameters in *bat-1*, *bat-2*, and *bat-3* mutants compared to WT controls.

### Metabolic crosstalk between GABA and flavonoid pathways

*CsBAT* regulates the levels of GABA, theanine, glutamine, and glutamate by coordinating or antagonizing the expression of key flavonoid pathway enzymes (LAR, 4CL, C4H). This interaction may stem from competition for chorismate-derived precursors or involve flavonoid derivatives (e.g. anthocyanins) modulating membrane permeability to influence *CsBAT* transport efficiency. Additionally, flavonoids’ antioxidant properties may affect the activity of GABA-synthesizing enzymes (e.g. GAD) [6, 25], revealing a coordinated network between secondary metabolism and amino acid transport. It is important to note that this study primarily reveals a transcriptional correlation. Future research involving metabolomic profiling to quantify catechins and functional genetic assays will be essential to definitively establish the regulatory role of CsBAT in flavonoid biosynthesis.

### Tissue-specific regulation of nitrogen redistribution

Silencing *CsBAT* led to significant amino acid accumulation in roots, while increasing amino acid levels in young shoots and decreasing them in mature leaves. This highlights *CsBAT*’s differential regulatory roles across tissues: In roots, *CsBAT* likely facilitates amino acid unloading into the phloem for long-distance transport via *CsGABA-T* and *CsGGT1*; silencing disrupts this process, causing amino acid retention. In mature leaves, high *CsBAT* expression may mediate senescence-associated amino acid redistribution to young shoots. Silencing reduces amino acid export from mature leaves, explaining the dynamic ‘source–sink’ shift in shoot amino acid levels.

As the dominant ‘sink’, tea shoots rely on nitrogen transported from roots and mature leaves, primarily in the form of amino acids [1, 2, 26]. This aligns with the physiological trait of young tea shoots depending on nutrient remobilization from mature leaves, underscoring *CsBAT*’s vital role in maintaining intertissue nitrogen homeostasis.

### Carbon flux reprogramming triggered by *CsBAT* silencing

*CsBAT* knockdown reversed the correlation patterns between flavonoid pathway genes (*PAL*, *4CL*) and amino acid metabolism. The C4H/PAL–amino acid relationship was inverted between RNAi and control groups, suggesting *CsBAT* indirectly modulates amino acid balance via phenylpropanoid metabolism. Phenylalanine accumulation (the flavonoid precursor) in RNAi tea lines may feedback-inhibit PAL activity or divert carbon/nitrogen resources, creating a negative correlation between flavonoid synthesis and amino acid metabolism. LAR’s coexpression with *CsBAT* implies that *CsBAT* may influence amino acid storage/transport in vacuoles by regulating proanthocyanidin synthesis (catalyzed by LAR). 4CL, a hub gene linking phenylpropanoid metabolism to lignin biosynthesis, showed negative correlations with amino acids, suggesting that *CsBAT* silencing redirects carbon flux from lignin to amino acid metabolism, thereby supporting shoot growth. 

## Materials and methods

### Plant materials

*Arabidopsis thaliana* seeds, wild-type (WT) (Col-0 ecotype), and T-DNA insertion mutants were cultivated in growth chambers (22°C, 50% relative humidity, 16-h light/8-h dark cycle; 120 μmol/m^2^/s light intensity). T-DNA insertion alleles *bat-1* (SALK_048889C), *bat-2* (SALK_048892C), and *bat-3* (SALK_107641C) were obtained from the *Arabidopsis* Biological Resource Center (ABRC). Homozygous mutants were screened by polymerase chain reaction (PCR) using gene-specific primers (*bat-1*-LP/RP, *bat-2*-LP/RP, *bat-3*-LP/RP) and the T-DNA left-border primer *LBb1* (Table S1). Transgenic lines were selected via antibiotic resistance, and four independent homozygous T_4_ lines were used for analysis. For *in vitro* assays, seeds were surface-sterilized with 12% (w/v) sodium hypochlorite for 15 min, rinsed three to four times with sterile water, and stratified on solid medium at 4°C for 3 days before transfer to growth chambers.

Tea plant (*C. sinensis* cv. ‘Shuchazao’) seeds from Dechang Tea Plantation (China) were sterilized, decoated, and germinated in vermiculite. Seedlings were transplanted to soil in growth chambers (conditions as above). Tissues (buds, stems, vascular bundles, leaves, and roots) from 8-year-old plants (Guohe Tea Plantation, Anhui, China) were flash-frozen in liquid nitrogen and stored at −80°C. The detailed procedure was as follows: stem vascular bundles and leaf main veins were isolated via manual microdissection from surface-sterilized tea samples (young shoots/stems/roots). After rinsing with sterile distilled water, tissues were disinfected in 70% ethanol (30 s) and triple-rinsed. Using sharp scalpels and fine forceps: (i) Stems were longitudinally split to expose vascular bundles; cortical/pith tissues were gently removed before meticulous dissection of target bundles. (ii) Leaves were flattened for lamina excision along main veins, preserving intact vein tissue (vascular bundle and surrounding sheath). All tools were kept sharp with minimized mechanical damage. Dissected tissues were immediately transferred to prechilled liquid nitrogen for RNA extraction.

For shade treatment, tea plants were covered with black shading nets allowing 20% light transmittance. Low-temperature treatment was conducted by exposing plants to 4°C for 3 days. For salt, abscisic acid (ABA), and polyethylene glycol (PEG 6000) treatments, experimental protocols followed Feng *et al.* [27]: plants were sprayed with 300 mM NaCl, 100 μM ABA, or 10% (w/v) PEG 6000. Uniformly grown tea cuttings were subjected to these stresses for 3 days. Subsequently, the second and third fully expanded leaves were collected, rinsed thoroughly with distilled water, snap-frozen in liquid nitrogen, and stored at −80°C for further analysis.

### Cloning and expression analysis of *CsBAT*

Total RNA was extracted using an RNA kit, assessed for quality (A260/A280: 1.9–2.1; agarose gel electrophoresis), and reverse-transcribed into cDNA (PrimeScript RT Kit, Takara, Japan). *CsBAT* expression was quantified via quantitative reverse transcription polymerase chain reaction (qRT-PCR), primers in Table S1) with *GAPDH* as the reference gene (2^−ΔΔCt^ method) [28]. Bioinformatics tools (DNAMAN, ProtParam, TMHMM, Phyre2) were used for sequence analysis.

### Vector construction and *Arabidopsis* phenotyping

The full-length open reading frame (ORF) of CsBAT was cloned into the plant overexpression vector pK7FWG2.0 [29] (Fig. S1) using Gateway™ technology (Invitrogen). This vector confers constitutive expression of the transgene under the control of the cauliflower mosaic virus (CaMV) 35S promoter and adds a C-terminal eGFP (enhanced Green Fluorescent Protein) tag to the resulting protein, enabling potential localization studies. The vector also contains a plant-selectable marker for kanamycin resistance. The construct (*35S:CsBAT-eGFP*) and the empty vector control (pK7FWG2.0) were introduced into *Agrobacterium* strain GV3101 via electroporation.

*Arabidopsis thaliana* homozygous T-DNA insertion mutants for the *AtBAT* gene (*bat-1*:SALK_048889C; *bat-2*: SALK_048892C; *bat-3*: SALK_107641C) in the Columbia-0 (Col-0) background were obtained from the ABRC. Floral dip transformation [30] was performed to introduce the *35S:CsBAT-eGFP* construct into the bat mutant lines for genetic complementation assays and into WT Col-0 plants for overexpression analyses.

Transgenic plants were selected in subsequent generations by growing seeds on half-strength Murashige and Skoog (one-half MS) medium supplemented with kanamycin (50 μg/ml). Homozygous T_4_ lines were used for phenotyping. For phenotypic evaluation, surface-sterilized seeds from homozygous transgenic lines, mutants, and WT controls were plated on modified MS media containing a range of nitrogen sources. After stratification at 4°C for 48 h, plates were placed in a growth chamber and grown vertically for 14 days under long-day conditions (16-h light/8-h dark cycle at 22°C). Phenotypic parameters, including rosette diameter, primary root length, fresh weight, and leaf count, were measured (*n* = 3 biological replicates, 4 seedlings/replicate).

### Chlorophyll fluorescence assay

Prior to experimentation, select clear weather conditions for measurements, ensuring the Handy PEA plant efficiency analyzer (PEA Plus, Hansatech) is fully charged and operational. *Arabidopsis thaliana* plants grown in soil for ~25 days should be selected, with healthy, intact, and clean green leaves used as test samples. For formal measurements, secure the target leaves in dark-adaptation clips, completely close the shutter for 20–30 min of dark adaptation, then immediately open the shutter and initiate chlorophyll fluorescence parameter measurements using the plant efficiency analyzer. Detailed records of plant identifiers and measurement sequences must be maintained.

Upon completion of experiments, import raw data into a computer and perform standardized processing using the instrument’s dedicated software (PEA Plus v2.1). Generate standardized OJIP curves and radar plots of fluorescence parameters for WT, mutant, and complementary lines, followed by systematic comparative analysis based on these visualizations.

### Subcellular localization

*Arabidopsis thaliana* leaves (5th–7th, prebolting stage, 3–4 weeks old) were used for protoplast isolation. (i) Protoplast preparation: Leaves were sliced into 0.5- to 1-mm strips and digested in enzyme solution (dark, 40 rpm, 2–3 h). The reaction was terminated with W5 solution, filtered through a 40-μm nylon mesh, and centrifuged (100× g, 8 min, 4°C). After supernatant removal, protoplasts were suspended in W5 and centrifuged. MMG solution was used to adjust protoplast density to 2 × 10^5^/ml. (ii) PEG-mediated transformation: 200 μl protoplasts were mixed with 10 μg plasmid (1–1.5 μg/μl), incubated for 5 min, then treated with 210 μl PEG4000 (gently mixed, RT, 5–30 min). After dilution with 800 μl W5 and centrifugation (100× g, 8 min), protoplasts were resuspended in WI solution and cultured overnight under low light. Staining with Dil (20 min) and DAPI (5 min) was followed by WI washing and centrifugation. Samples were resuspended in 100 μl WI and observed under confocal microscopy (405/488 nm excitation).

### GABA transport assays in yeast

The ORF sequence of *CsBAT* was cloned into the pYES2 yeast expression vector using primers designed with appropriate restriction sites. Purified PCR products were ligated into pEASY-Blunt for sequencing, then subcloned into pYES2 (Fig. S2). Recombinant plasmids were transformed into 22Δ10α yeast competent cells.

WT (strain 23344c), empty vector (22Δ10α-pYES2), and CsBAT- expressing (22Δ10α-CsBAT) yeast strains were cultured in YNB medium (2 mM ammonium sulfate) to log phase (OD600 = 0.8). Cells were pelleted (4000× g, 5 min, 4°C), resuspended in nitrogen-free YNB, and starved for 2 h (30°C) to induce nitrogen deficiency. (i) Kinetic analysis: Six GABA concentrations (0.09375–8 mM) were tested, with sampling at 0, 2, 5, 10, and 20 min (*n* = 3). Reactions were stopped with ice-cold phosphate-buffered saline (PBS). (ii) Substrate specificity: 200 μM GABA was co-incubated with 2 mM competitors (theanine, glutamate, glutamine, valine, aspartate, or alanine) for 10 min. (iii) pH dependence: Reactions (200 μM GABA, 10 min) were conducted at pH 4.0–8.0. (iv) H^+^-pump inhibition: 200 μM GABA was treated with Carbonyl cyanide m-chlorophenylhydrazone (CCCP, 0.01 mM), 2,4-Dinitrophenol (DNP, 0.1 mM), or Diethyl pyrocarbonate (DEPC, 1 mM) for 10 min.

Cell pellets were washed four times with cold buffer (0.6 M sorbitol, 50 mM potassium phosphate, pH 4.5). Theanine was extracted by boiling in 1 ml H_2_O (98°C, 1 h), filtered (0.22 μm), and lyophilized. GABA/theanine were quantified using a commercial kit (Beijing Boxbio Science & Technology Co. Ltd.) and HPLC (Waters 2695; AccQ·Tag column). Mobile phases: (i) 140 mM sodium acetate (pH 6.4), (ii) acetonitrile (1 ml/min, 37°C, 248 nm). Data (mean ± SD, *n* = 3) were analyzed by one-way analysis of variance (ANOVA, SPSS 22.0).

### RNAi hairy root transformation

A 700-bp gene-specific fragment of *CsBAT* was amplified and cloned into the RNA interference (RNAi) vector pCAMBIA1301. The resulting construct was then transformed into *Agrobacterium rhizogenes* strain A4. Positive bacterial transformants were preinduced with acetosyringone and used to infect root-excised, 3-month-old tea seedlings (cultivar ‘Shuchazao’, possessing 3–4 true leaves) following established methods [31, 32]. Briefly, the plants were wounded and inoculated with the bacterial suspension. After cocultivation in darkness, the plants were transferred to a hormone-free medium supplemented with cefotaxime (100 mg/l) to eliminate *Agrobacterium*. Emerging hairy roots were screened via PCR using vector-specific primers to confirm transformation. Transgenic hairy roots exhibiting differential *CsBAT* expression levels were subsequently selected for qPCR analysis and amino acid profiling using the Waters AccQ·Tag method.

### *In situ* hybridization

The spatial localization of CsBAT in young leaves, stems, and roots of *C. sinensis* cultivar ‘Shuchazao’ was analyzed using the RNA *in situ* hybridization protocol described by Wang *et al*. [33]. Tissue samples were fixed in FAA solution (50% ethanol, 5% glacial acetic acid, 3.7% formaldehyde) overnight, followed by gradient dehydration using a Leica ASP200 automated tissue processor (Leica Microsystems, Germany). Dehydrated samples were paraffin-embedded and sectioned at 10 μm thickness with a rotary microtome (Leica Reichert–Jung, Wetzlar, Germany).

Sections were mounted on 3-aminopropyltriethoxysilane (APTES)-coated slides (Thermo Fisher Scientific, Waltham, MA, USA) and hybridized with digoxigenin (DIG)-labeled sense or antisense riboprobes. The DIG-labeled probes were synthesized following the manufacturer’s protocol (Roche Applied Science, Penzberg, Germany), targeting the nonconserved 5′-untranslated region (UTR) of CsBAT to minimize cross-hybridization with other transporter genes. Key experimental steps included: Rehydration: Sequential treatment with xylene, absolute ethanol, graded ethanol series, and PBS buffer; RNA unmasking: Proteinase K digestion (37°C, 30 min) followed by termination with 2 mg/ml glycine-PBS solution; Hybridization detection: Overnight hybridization with specific probes at 55°C, with subsequent DIG-antibody-based signal detection.

### Data analysis

Data are mean ± SD (*n* = 3). ANOVA and Duncan’s test (*P* < 0.05) were performed in SPSS 22.0. Kinetic parameters were derived from Michaelis–Menten plots (GraphPad Prism 8.0).

## Supplementary Material

Web_Material_uhaf261
