# Supplementary figures and images for: Amino acid transporter CsBAT links GABA accumulation to flavonoid metabolism in Camellia sinensis
Source: Hortic Res. 2025 Oct 1;13(1):uhaf261. doi: 10.1093/hr/uhaf261 (PMC12861478; doi:10.1093/hr/uhaf261)

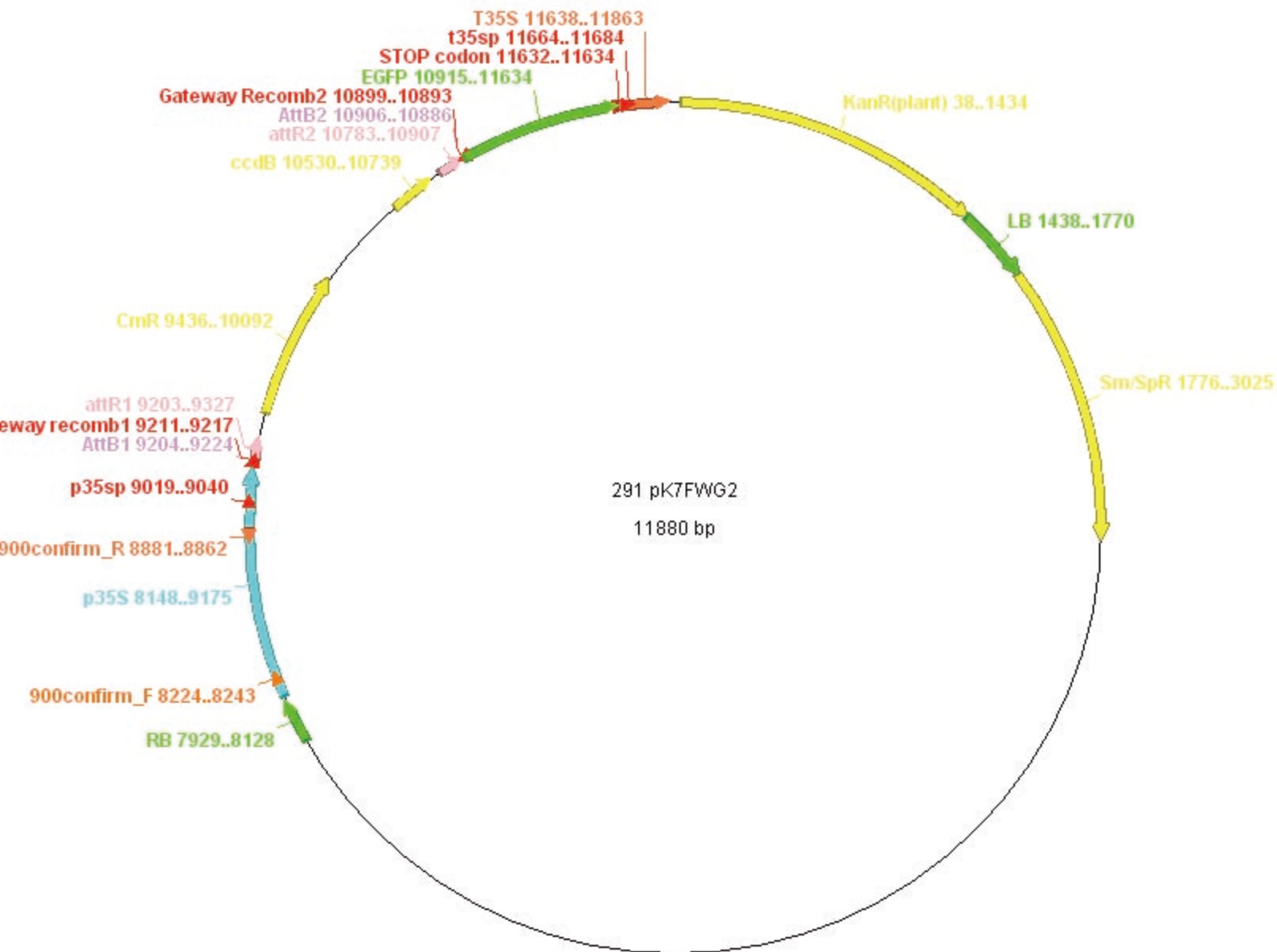

Supplement: Web_Material_uhaf261 [file web_material_uhaf261.zip › Figure S1.pdf]

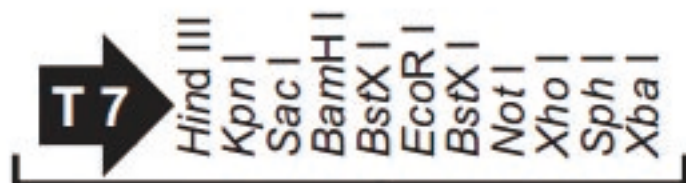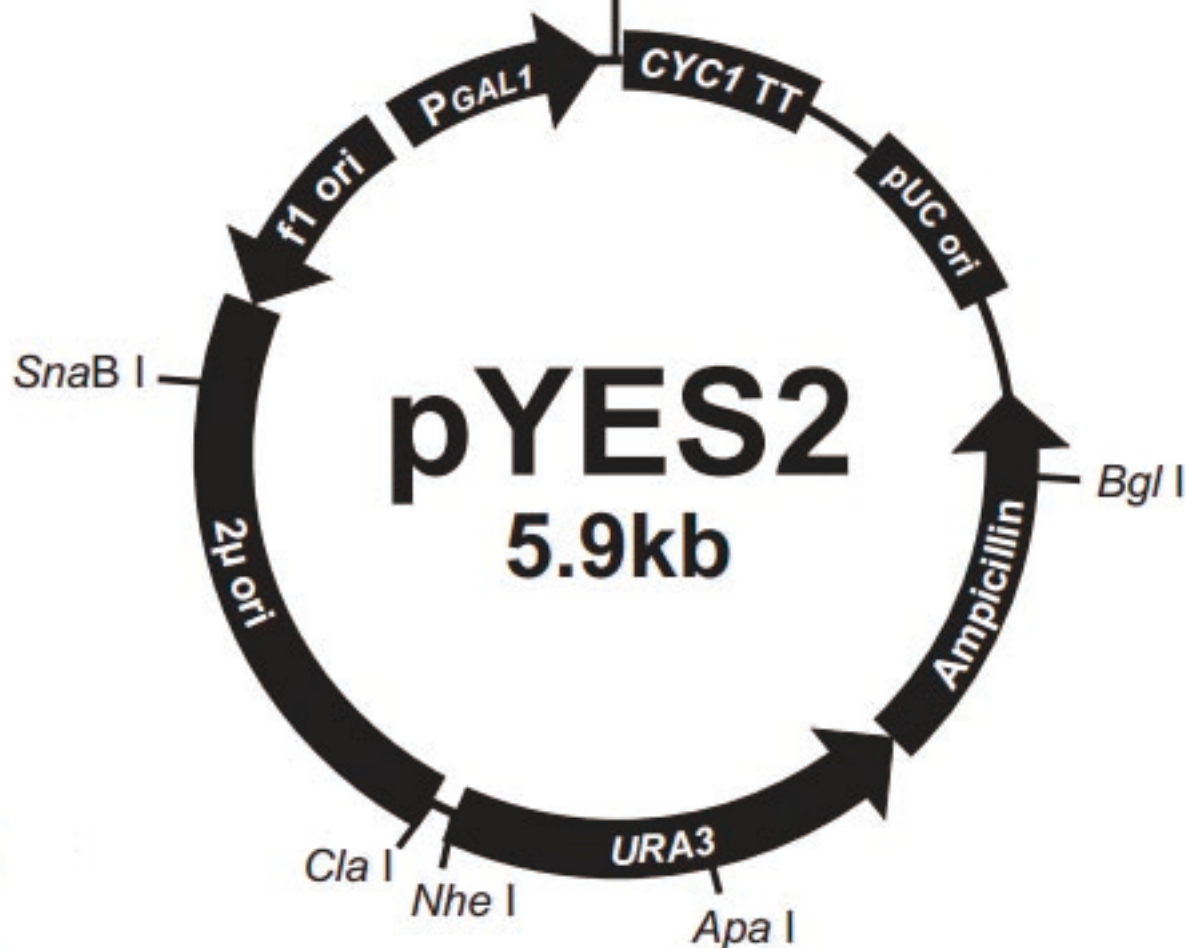

Supplement: Web_Material_uhaf261 [file web_material_uhaf261.zip › Figure S2.pdf]
