# Supplementary material for: Amino acid transporter CsBAT links GABA accumulation to flavonoid metabolism in Camellia sinensis
Source: Hortic Res. 2025 Oct 1;13(1):uhaf261. doi: 10.1093/hr/uhaf261 (PMC12861478; doi:10.1093/hr/uhaf261)
Supplement: Web_Material_uhaf261 [file web_material_uhaf261.zip › Table S1.doc]

Table S1. List of primers used in this study

| Genes | Forward primers 5′→3′ | Reverse primers 5′→3′ |
| --- | --- | --- |
| **Primers in *Arabidopsis* plants** | | |
| *bat-1*-LP/RP | ATTTCGCAAACACGTAAGGTG | AAGTTCCCCAATCAAAATTGG |
| *bat-2*-LP/RP | AGACTCGTATCCTCCTTTGCC | AGTTAAAACGGATTGAACCGG |
| *bat-3*-LP/RP | GAAGAGACAGTCGACGCAGAC | AGCTCAGTTGGTTAGAGCGTG |
| GateBAT | GGGGACAAGTTTGTACAAAAAAGCAGGCTTCATGGGATGGCACTCCCAT | GGGGACCACTTTGTACAAGAAAGCTGGGTTCACAAAACATCGTCTAT |
| CsBAT | TTAAGCTTGGTACCGAGCTCATGGGATGGCACTCCCATTCTCA | CGTTACTAGTGGATCCTCACAAAACATCGTCTATGTTGGTGAT |
| **Primers for Gene Cloning in Yeast** | | |
| CsBAT | GGATCCATGGGATGGCACTCCCATTCTCA | CTCGAGTCACAAAACATCGTCTATGTTGGTGAT |
| **Probe for *in situ hybridization*** | | |
| Sense-probe | GAGCGAGAACAAUGAUGCCGGUGGA |  |
| Anti-sense-probe | UCCACCGGCAUCAUUGUUCUCGCUC |  |
| **Primers in qRT-PCR** | | |
| CsBAT | ACAGCTCACTCGATACCGGCCATAC | CACCAGCAATAAGCCACCCATAAAC |
| CsGDH | GAGCTGAAGACATACATGACCA | GCACGAGCAACACGATTAA |
| CsGAD | AGTGACATCCAGAAAGTCTTGCT | CACCATTAGTCTTCTTCCTACTGAG |
| CsGABA-T | TTCACAGATAACAAGTCACCTAAT | TCTCACACTCTGCTCCAA |
| CsSSADH | TGCAGGATTAGCTGCTTATGT | CGAGGCCAGACTGCTTAAA |
| CsADC | GTTTCGGGTGGAGCATACAA | TCAGCATAGAGCAAGCAAGTATC |
| CsSAMDC | GATGGGTTCAGTTACGCTAGTT | CACAGTAGTATCCCTGCACATC |
| CsTS | AGACCGCCGACATCAACAC | ATGGCTTCCACAGCAGAGT |
| CsGS | ATCAGTTGTGGATGGCTCG | CACTTCGCATGGACTTGGTAC |
| CsGGT | GATGAATCTTGGTGATCCTGAT | TTCCACCGTCCACCATAGT |
| CsF3H | ATCCTCTCTAAACCCCTCC | ATCCCACAAGTGCCCAT |
| CsCHS | CATTACTAACAGCGAGCATAAAACG | TAGTTTTGGGACTTCAACAACCAC |
| CsPAL | ATGACTTCTACAACAATGG | GGAGTTCTGAGCAATAAG |
| Cs4CL | CGTGGTCCTCAAATTATG | CATCATCGTCGTCTACAT |
| CsC4H | CAATGGCAATGACTTTAG | CTCAGCAGTATCAATCTT |
| CsDFR | AGTTGTGTCGTTCTCATC | GTATCAATGGCTCCTCTG |
| CsLAR | GGGGCATCCTGTATCAAAGA | CCGCATACCTTTCAGTCCAT |
| CsANR | GCGAAGTTGATCCTCTCGTC | AACCACATCGTCAAGTGAACA |
| CsGAPDH | TTGGCATCGTTGAGGGTCT | CAGTGGGAACACGGAAAGC |
